# Screening for carriage of carbapenem-resistant Enterobacteriaceae in settings of high endemicity: a position paper from an Italian working group on CRE infections

**DOI:** 10.1186/s13756-019-0591-6

**Published:** 2019-08-13

**Authors:** Simone Ambretti, Matteo Bassetti, Pierangelo Clerici, Nicola Petrosillo, Fabio Tumietto, Pierluigi Viale, Gian Maria Rossolini

**Affiliations:** 1 Unit of Clinical Microbiology, St Orsola-Malpighi University Hospital, Bologna, Via Massarenti 9, 40138 Bologna, Italy; 2 Infectious Diseases Clinic, Department of Medicine University of Udine and Azienda Sanitaria Universitaria Integrata di Udine, Udine, Italy; 3 Microbiological Unit, Department of Laboratory Medicine and Diagnostics Biotecnology, Azienda Socio Sanitaria Territoriale Ovest Milanese, Via Giovanni Paolo II, 2025 Legnano, Mi Italy; 4 National Institute for Infectious Diseases “L. Spallanzani”, IRCCS-, Rome, Italy; 5 Infectious Diseases Unit, Department of Medical and Surgical Sciences, S.Orsola-Malpighi Hospital, University of Bologna, Via Massarenti 9, 40138 Bologna, Italy; 6 Department of Experimental and Clinical Medicine, University of Florence, and Clinical Microbiology and Virology Unit, Florence Careggi University Hospital, Florence, Italy

**Keywords:** Carbapenem-resistant, Enterobacteriaceae, Active surveillance, Infection control

## Abstract

**Introduction:**

A variety of national and international guidelines exist around the management of carbapenem resistant Enterobacteriaceae (CREs), but some of these are several years old and do not reflect current epidemiology and they also do not necessarily give pragmatic advice around active surveillance of CREs in countries with a high burden of cases and limited resources. This paper aims to provide a best practice position paper to guide active surveillance in a variety of scenarios in these settings, and discusses which patients should be screened, what methods could be used for screening, and how results might influence infection prevention interventions.

**Methods:**

This paper was developed as a result of a series of meetings of expert opinion leaders representing the major infectious disease and infection prevention societies in Italy and having the endorsement of AMCLI (Italian Association of Clinical Microbiology) and SITA (Italian Society for Anti-infective Therapy). There was no attempt to undertake a full systematic review of the evidence, as it was felt that this was inadequate to inform a pragmatic view on the best way forward based on current epidemiology and infection rates.

**Key recommendations:**

Key recommendations focus on the urgent need to promote measures to prevent transmission and infection, focusing on high risk patients and clinical areas, as well as outbreak situations. Active surveillance leading to appropriate infection prevention precautions plays a major role in this.

**Conclusions:**

There are limited national or international guidelines giving pragmatic advice on the most appropriate measures for active surveillance and management of colonized patients in a high-burden setting such as Italy. While individual hospitals and regions will need to formulate their own policies based on local epidemiology, this position paper attempts to highlight current best practice in this area and provide pragmatic advice for clinicians, infection prevention staff, and healthcare managers.

## Background

### Aims and target groups

The purpose of this paper was to address practical questions for clinicians, infection prevention and control (IPC) practitioners, and diagnostic laboratory personnel dealing with the screening of carbapenem-resistant Enterobacteriaceae (CRE) carriage in healthcare settings from areas of high CRE endemicity, in view of the fragmentation in the observational literature on this topic and the lack of randomized clinical trials.

## Methods

A review of the current literature regarding the situation with CRE in Europe, with a focus on Italy as an area of high endemicity, and of the current laboratory methods for screening for CRE carriage, were undertaken. Subsequently, a panel of seven experts developed a list of questions to be addressed in the paper, with three questions being formulated after three rounds of discussion among panel members. In view of the lack of strong evidence, panel members were asked to provide narrative answers on the basis of their knowledge and experience in the field. Finally, answers were reviewed and discussed by the panel, until a consensus was reached.

### CRE: definitions and mechanisms

CRE are among the most challenging antibiotic-resistant pathogens emerged in the clinical setting, due to their ability to spread rapidly in healthcare environments and to cause infections associated with high morbidity and mortality, for which very limited treatment options are available [1].

At least two different mechanisms can be responsible for the carbapenem-resistant phenotype of CRE strains, including: i) decreased outer membrane permeability in combination with the overproduction of β-lactamases with marginal carbapenem-hydrolyzing activity (e. g. some AmpC-type or extended-spectrum β-lactamases); and ii) the production of β-lactamases with efficient carbapenemase activity (carbapenemases). The former mechanism is dependent on chromosomal mutations: mutants are usually selected under carbapenem treatment and encountered sporadically or causing small outbreaks, and usually exhibit only a moderate increase of carbapenem minimum inhibitory concentration (MIC) [2]. Carbapenemase production is due to the horizontal acquisition of one or more mobile carbapenemase genes (usually plasmid-borne) and is the most prevalent and epidemiologically relevant resistance mechanism. In fact, in settings of high CRE prevalence, carbapenemase-producing Enterobacteriaceae (CPE) usually contribute the majority of CRE isolates from clinical sources [3].

The relationship between CRE and CPE is one of broad but not complete overlapping, since most but not all CRE are CPE and vice versa. In fact, some CRE are not CPE (i. e. those with a carbapenem resistance mechanism other than carbapenemase production) and some CPE are not CRE (i. e. those which exhibit low carbapenem MICs and remain phenotypically susceptible to carbapenems).

Several different carbapenemases can be encountered among CPE, including class A serine β-lactamases (e. g. KPC-type enzymes), class D serine β-lactamases (e. g. OXA-48 and related enzymes) and class B metallo-β-lactamases (e. g. NDM-, VIM- and IMP-type enzymes). Carbapenemases of different classes exhibit different functional properties, which may be relevant to their phenotypic detection (e. g. KPC enzymes are inhibited by boronic acids but not by EDTA and can be detected by synergy testing using these inhibitors) and to clinical treatment (e. g. KPC enzymes are inhibited by the new β-lactamase inhibitors avibactam and vaborbactam, while this is not the case for the metallo-β-lactamases) [4].

### Diversity of CRE epidemiology in Europe, and the Italian perspective

In Europe, CRE have been reported from most countries, although with variable prevalences. In many countries the overall prevalence remains low, with most cases being related to cross-border transfer followed by occasional outbreaks. However, in a few European countries (e. g. Greece, Italy, Romania) CRE have experienced a massive dissemination leading to a condition of high-level endemicity, while in some other countries (e. g. Spain, Portugal, Bulgaria) the prevalence of CRE is lower but reported on the increase. The epidemiological diversity among different European countries does not only concern the overall prevalence of CRE but also the type of prevalent carbapenemases among CPE, as shown by the results of the recent EuSCAPE survey [5].

Italy has been among the first European countries to report the isolation of CPE from clinical specimens [6, 7], and is one among the countries which have experienced a rapid and massive dissemination of CRE since 2010. In Italy, this CRE epidemic has mostly been contributed by the dissemination of *Klebsiella pneumoniae* strains producing KPC-type carbapenemases (KPC-Kp), while CRE strains producing other carbapenemases or those not producing carbapenemases have remained relatively uncommon [8, 9]. In particular, despite their earlier emergence, VIM producers have remained sporadic or at most were associated with small outbreaks. On the other hand, strains producing NDM-type or OXA-48-like carbapenemases were reported more recently, as sporadic cases or causing small outbreaks, usually associated with cross-border importation [10–14]. Recent nationwide surveillance data show that KPC represents 95% of carbapenem resistance genes in *E.coli* and *K pneumoniae* strains isolated from bloodstream infections [15]. This epidemiological scenario is different from that of other European countries of high CRE endemicity. For instance, Greece experienced a remarkable diffusion of VIM-producing strains since the early 2000s, and thereafter this scenario was compounded by the superimposition/partial replacement with KPC-Kp and, more recently, also with OXA-48-producers [16, 17].

In Italy, the CRE epidemic started since 2010 rapidly expanded at a countrywide level, and CRE are currently encountered not only among inpatients from acute-care hospitals but also among outpatients [18] and patients from long-term-care facilities [19, 20]. Reported prevalences of CRE carriage were variable in different settings, also reflecting different patient populations, screening strategies and study periods. Prevalences ranging from 0.2 to 3.9% were reported among inpatients from acute care hospitals in northern Italy, at the beginning of the CRE epidemic [21, 22]. Very high rates of CRE carriage (28.4%) were recently reported among patients from a long-term acute-care rehabilitation facility (LTACRF) in central Italy [20], underscoring the role that LTACRF can play in the dissemination of CRE in endemic areas.

### The burden of CRE

The archetype of a CRE target is a patient affected by many comorbidities, with a history of repeated hospital admissions, long hospital stay and undergoing relevant management complexities. Consequently, the higher the complexity of the care pathway and the fragility of the patient, the greater the likelihood of CRE colonization and subsequent invasive disease [23]. Regarding invasive diseases, bacteremia, pneumonia and surgical site infections are an increasingly complex challenge in relation both to the predicted poor patient clinical status and scanty therapeutic options [24, 25].

In a recent meta-analysis, an overall 16.5% risk of infection with CRE amongst patients colonized with CRE has been suggested [26]. However, rates of infection reported in individual studies varied widely, from 0 to 89%, mainly in relation to case mix and comorbidities, with the majority falling in the range of 7.6–44.4% [27, 28].

In colonized patients, chemotherapy for acute leukemia, solid organ transplantation, and ICU stay represent the most important patient-related conditions associated with a significant risk of CRE infections [29]. Likewise, prolonged exposure to broad spectrum antibiotics and presence of central venous catheters are the main modifiable variables involved. A recently published case-control study of long-term acute care hospitals (LTACH) patients found that independent factors associated with CRE colonization and infection in this setting included solid organ and stem cell transplantation, mechanical ventilation, fecal incontinence, and exposure in the prior 30 days to carbapenems, vancomycin, and metronidazole [30]. Finally, CPE are associated with considerable mortality, and they may be more virulent than non-carbapenemase-producing CRE and are associated with poorer outcomes [31].

Costs of the diseases sustained by multidrug resistant (MDR) pathogens are well known [32, 33]. Of particular concern is the clinical and economic impact of MDR gram-negative bacilli because of the substantial changes in workflow and processes required [34]. Moreover, in the case of CRE, costs could be higher than with other resistant Gram-negative bacteria because they are more difficult to treat and need more extensive prevention and aggressive control activities. Indeed, many elements contribute to the real costs associated with the control of infections and colonization supported by CRE. These include the costs associated with the need for increased surveillance within each hospital to determine which pathogens are problematic by patient type and hospital care.

The first steps to control the spread of CRE infection consist of implementing a multimodal strategy for the management of carriage, including early identification, contact precautions, patient isolation, hand and environmental hygiene, and antimicrobial stewardship (see below). This strategy directed at interrupting cross-transmission, is considered the most cost-effective in any economic analysis regarding multidrug resistant organisms (MDRO). A recently published review [35] has noticed that the costs calculation is highly variable across published studies, mainly because of the high heterogeneity in methods, organism, involved country and hospital.

Recently, an estimate of the costs associated with an outbreak of carbapenemase-producing Enterobacteriaceae (CPE) was performed during an outbreak of New Delhi metallo-beta–lactamase producing *Klebsiella pneumoniae* in a group of five hospitals in London, with approximately 1500 beds and 190,000 admissions per year. Costs associated with the outbreak were split into actual expenditure (enhanced and enlarged screening activities, additional infectious disease consultant time, expenditure for antimicrobials, contact precautions, isolation costs, monitoring of hand washing, environmental decontamination, potential bed closures) and ‘opportunity cost’ (including additional staffing time, missed revenue and extended length of hospital stay). The overall expenses related to the outbreak were around € 1.1 million over 10 months (range 0.9–1.4 million), composed of € 312,000 for actual expenditure, and € 822,000 (range 631,000–1.1 m) for opportunity cost [36].

The Centers for Disease Control and Prevention have developed a CRE clinical and economic outcomes model to determine the cost of CRE related disease, by infection site including bloodstream infections, pneumonia (ventilator associated or not), complicated intra-abdominal infections and complicated urinary tract infections. Costs were considered from a hospital, a third-party payer and a social perspective. The first included costs related to the additional length of stay attributable to CRE infection, the second consisted of direct costs for hospitalization, treatment and associated diagnostic tests, while the social perspective included direct and indirect (i.e. productivity losses and mortality) costs. Taken as a whole the cost for every case could be quantified as up to 181,164 USD depending on infection type, contributed by up to 66,031 USD for the hospital, up to 31,621 USD for third-party payers, and up to 83,512 USD for society [37].

It is therefore evident that the cost of CRE infections is higher than the annual cost of many chronic diseases and of many acute diseases, and these could further increase if we consider the rising legal expenses, related to lawsuits. Given the growing epidemiological threat in Italy, as in several other European countries, investment in prevention and control are urgent as well as mandatory.

### Control strategies for CRE, and roles of active screening for detection of CRE carriage

In 2013 a group of experts under the auspices of the European Society of Clinical Microbiology and Infectious Diseases (ESCMID) performed a systematic review of the articles published on IPC measures for preventing the spread of multidrug-resistant organisms in the healthcare setting, in order to determine their effectiveness and to define the indication for application for specific types of resistant strains [38]. Moreover, IPC measures were studied according to the epidemiological setting, i.e. endemic or epidemic. An endemic setting was defined as a setting where there are constant challenges from admission of patients colonized or infected by multidrug-resistant organisms. An epidemic setting was defined as a setting with an unexpected increase of cases of infection by multi-drug resistant organisms or emergence of new strains not previously isolated in that setting. The GRADE methodology was applied for defining quality of evidence and strength of recommendations. Eighty-six studies from 1981 to 2011 were included in the final analysis. The main IPC measures and other strategies that were evaluated included hand hygiene, contact precautions, active screening cultures, environmental cleaning, antimicrobial stewardship, decolonization, and infrastructure and education. Both in endemic and in epidemic settings, implementation of hand hygiene education programmes was considered a strong recommendation to reduce the transmission of ESBL-producing Enterobacteriacae and multidrug-resistant gram negative organisms, with different levels of evidence from moderate to very low. The effectiveness of contact precautions for preventing the transmission of multidrug resistant Gram-negative organisms is controversial. In the epidemic setting there is a strong recommendation for implementing contact precautions for all colonized and/or infected patients. Moreover, there is a strong recommendation for using alert codes to identify known colonized patients at admission and perform screening and pre-emptive contact precaution. Isolation in a single room both for colonized and infected patients represents a strong recommendation together with cohort staffing (only for MDR- *K. pneumoniae*). In the endemic setting, implementation of contact precautions for patients colonized with MDR resistant gram negative organisms represents a strong recommendation. Regarding one of the more controversial points in everyday practice, i.e. the active screening for detection of CRE carriage, in the epidemic setting the ESCMID guidelines strongly recommend the implementation of active screening for the main resistant gram negative pathogens at hospital admission followed by contact precautions; regarding the endemic setting, due to lack of evidence, active screening should be suggested only as an additional measure. Monitoring cleaning performance to ensure proper environmental cleaning represents a strong recommendation both in the epidemic and in the endemic setting. Finally, antimicrobial stewardship and education are also strongly recommended in both settings.

A following article evidenced some controversies in the recommendations from various sources, including ESCMID, Irish, English, Scottish and ECDC guidelines reflecting the poor quality of the evidence base. These controversies include when to apply contact precautions, single room isolation, active screening culture, staff and patient cohorting, healthcare workers’ screening, patient decolonization and environmental cleaning [39].

In 2016 ECDC published documents on the rapid risk assessment of carbapenem-resistant Enterobacteriaceae and *Acinetobacter baumannii* in healthcare settings [40, 41]. The main options for actions to reduce identified risks included proper and timely clinical management, infection prevention and control measures in hospital and other healthcare settings, active screening in the epidemic setting and active surveillance in the endemic one, and antimicrobial stewardship. For CRE, a major emphasis was given to targeting patients at high risk for carriage of CRE, i.e. patients who had recently been hospitalised in a country or a region known as having a high CRE prevalence, or who were transferred from a hospital with high CRE prevalence. These patients should be screened for CRE digestive tract carriage and pre-emptive contact precautions and isolation should be considered.

The report from ECDC concerning the effectiveness of infection control measures to prevent transmission of CPE stated that no study assessed screening/surveillance as a single intervention, but the authors concluded that there is evidence to suggest that active rectal screening/surveillance on admission to hospital or a specific ward and during an outbreak can effectively limit and prevent the spread of CPE. The ECDC document recognizes, among other most effective interventions, patient isolation, patient cohorting and application of contact precautions. All these measures are usually applied when a patient is diagnosed as colonized and/or infected by CPE.

In Italy, the first agency to produce a document for the control of CRE was the Region Emilia Romagna in 2012 [42]. The infection control measures recommended to the healthcare settings in the region included phenotypic confirmation of carbapenemase production, active surveillance of asymptomatic carriers and contact isolation precautions for carriers. Surveillance of asymptomatic carriers was performed by rectal swabs for close contacts of hospitalised patients with CPE (patients staying in the same hospital unit), high-risk patients at hospital admission (i.e. patients transferred from other acute hospitals and LTCFs or coming from endemic countries) and, only for hospitals where CPE were endemic (with sustained intra-facility transmission) or where epidemic clusters were detected during the previous year, patients admitted to intensive care units, spinal cord injury units, transplant units, oncology and hematology units. CPE screening for carriers was not recommended in LTCFs.

Subsequently, the Italian Ministry of Health produced an Act [43] for the implementation of national surveillance of bloodstream infections by carbapenem resistant *Escherichia coli* and *Klebsiella pneumoniae.* The Act recommends active screening in all contacts of CRE-positive patients, in all patients with a previous colonization/infection that are admitted to hospital, and in all patients coming from endemic areas. Moreover, screening was suggested for patients admitted or transferred to high-risk units and in patients transferred from another hospital or with a history of recent hospitalization or coming from long term care facilities. Ministry of Health recommended also contact precautions and isolation for all colonized/infected individuals, including cohorting strategies, strengthening hand hygiene procedures and education.

Since CRE colonization is associated with an increased risk of CRE infection, knowledge of CRE colonization can be relevant not only to infection control but also to antimicrobial stewardship. In particular, in certain categories of colonized patients who are at high-risk for invasive infections (e. g. neutropenic patients), knowledge of CRE colonization can be relevant to the selection of empiric antimicrobial chemotherapy covering the colonizing pathogen in case of emergence of a septic status In this case, knowledge of the resistance mechanism of the colonizer is particularly important, given the different spectrum of activity of anti-CRE agents for example, the empirical use of ceftazidime-avibactam could be considered in case of a CPE producing KPC but not in case of a CPE producing a metallo-β-lactamase [44].

### Methods for active screening of CRE carriage

Screening for CRE colonization is usually based on microbiological evaluation of rectal swabs. To date there is little evidence that swabs from alternative sites could add more useful information, although screening from any other site putatively colonized could be considered. The isolation of CRE from other sites normally screened for colonization by MDR pathogens in ICU patients (i.e. nasal swabs, pharyngeal swabs, bronchial aspirates, urine cultures in catheterized patients) should always be considered and carefully evaluated in at-risk patients. CRE isolation from any clinical site should dictate an indication for a rectal swab, because invasive disease or colonization in the absence of intestinal carriage may represent a break in the standard of health care workers behavior and infection control procedures [45].

Surveillance tests to detect CRE present three main challenges: turnaround time (TAT), sensitivity and specificity. Screening methods can be subdivided into two major groups:

1. culture-based methods;

2. nucleic acid amplification technology (NAAT)-based assays.

#### Culture-based methods for CRE screening

Culture-based methods have been widely used for CRE screening, largely because initial testing is relatively easy to implement, as the necessary equipment and knowledge are already present in routine microbiology laboratories. Several different cultural approaches have been described:

a) inoculation onto McConkey agar plate after broth enrichment (CDC method) [46];

b) direct inoculation onto McConkey agar plate containing a meropenem disk [47];

c) direct inoculation onto specific selective chromogenic media [48–50].

The CDC screening method addresses the need to maximize sensitivity (detection of low-level resistance and/or low loads of CRE), but recent reports [48, 49] showed that other culture-based protocols have a better or at least comparable performance. Moreover, the CDC method has significant limitations: in particular, the TAT for a positive or preliminary positive result is slow (48–72 h) and the protocol is time-consuming for the laboratory workflow.

The main advantages of direct inoculation onto McConkey agar plate containing a meropenem disk include low cost, ease of evaluation of suspected colonies and the possibility of checking the quality of the samples (low quality, if no bacterial growth is observed). Some authors suggested the use of a second disk, containing meropenem and boronic acid, to rapidly discriminate the presence of KPC-producing isolates. TAT for positive samples is typically 24–48 h. However, the need to add the meropenem disk after inoculum represents a significant increment of manual workload and also a risk of contamination. A main concern with this method is lack of sensitivity, especially for CPEs which typically show MICs to the carbapenems at or around the breakpoint [48].

A number of selective chromogenic media have been developed to simplify culture-based protocols [48–50]. These media usually incorporate a carbapenem as selective agent and substrates resulting in color change when hydrolyzed by Enterobacterales. Advantages of this approach include an easy workflow for inoculation and evaluation of growth, presumptive species identification, good sensitivity and specificity. The TAT for positive samples is 24–48 h. However, selective media that were specifically designed to target KPC producers have shown low sensitivity for mechanisms due to other enzymes, particularly OXA-48 [51]. Moreover, selective chromogenic media are more expensive that McConkey agar and, when using the former media, a second non-selective plate should ideally be inoculated in parallel to check for the quality of the rectal swabs.

It is noteworthy that all culture-based methods only allow the identification of CRE in general, but a positive result requires further evaluation to confirm carbapenemase production [52, 53]. The turnaround time for confirmatory testing may range from a few minutes to 24 h, depending on the methods chosen, with variable additional cost. Hospital infection control programs can act on negative results and preliminary positive results, pending confirmation, but it is obvious that the final reporting time (TAT of screening + TAT of confirmation test) could have an important impact on infection control performance. Despite these limitations, culture-based methods still maintain some peculiar advantages. First of all, they can detect all types of CRE, including organisms producing not previously known carbapenemases. Moreover cultural methods are fundamental to recover viable organisms, allowing the performing of phenotypic antimicrobial susceptibility testing, the collection and storing of CRE strains.

#### NAAT for CRE screening

Molecular based methods (NAAT) for CRE screening usually detect the presence of one or more carbapenemase genes. For this reason, these assays are able to identify only previously known resistance determinants.

Reliable detection of multiple genes, as well as extraction, could represent a challenge for NAAT applied to high-complexity samples such as stool or rectal swabs. Another critical issue is related to the typical feature of CPE epidemiology: since global CPE diffusion is complex and typically shows differences between different countries, molecular assays suitable for screening use should cover a broad spectrum of carbapenemase genes, including at least the five most common families (KPC, VIM, NDM, IMP, OXA-48).

Despite these difficulties, several in-house and commercial molecular methods for CPE/CRE surveillance have been developed in the last few years with the aim to overcome most of the limitations of culture-based methods and, in particular, to reduce TAT. NAAT-based assays validated for carbapenemase genes detection from rectal swabs can also be used as a confirmatory test for suspected colonies identified by culture-based methods, although not all commercial assays have an on-label indication for this.

Briefly, three types of NAAT-based assays can be taken in consideration for CPE/CRE surveillance: in house molecular methods, commercial molecular assays, and rapid/easy to use commercial molecular assays.

In house molecular methods can show good level of sensitivity and specificity [54]. Moreover, these assays are less expensive if compared to molecular commercial methods; on the other hand, important disadvantages are low level of automation, standardization and validation, and suboptimal inter-laboratory reproducibility.

Commercial molecular assays are highly sensitive, specific and standardized, with TAT of few hours; the level of automation of these methods can vary from poor (need of sample preparation step, including extraction or lysis, and/or multiple hands-on steps), to good, but for all these assays laboratory experience and equipment are required.

Rapid/easy to use commercial molecular assays (REU-CMA) may provide the same high standard of quality of results [55], with shorter hands-on time and TAT (less than 1 h) and no requirement for batching. Furthermore, these fully automated methods can be used as point of care testing, streamlining the diagnostic workflow in particular if the microbiology lab is distant from the care structure or during closing hours. However, coverage within carbapenemase gene families, level of automation, turn-around time and suitability as point of care testing widely vary between different commercial molecular methods. It should be noted that time to result and easiness of access to results are particularly relevant issues for infection control purposes.

Currently, limitations of NAAT assays include laboratory acquisition costs and the ability to detect a limited number of known carbapenemases, while the main advantages are the short TAT for both negative and positive results that could positively impact at the organisational level on infection control programmes, and the improved sensitivity over some cultural methods. Moreover, as noted previously, awareness of the specific carbapenemase detected can be critical in decisions around optimal antimicrobial therapy.

### Strategies for active screening of CRE carriage in settings of high-level endemicity

Several strategies can be considered for active screening of CRE carriage in healthcare settings, depending on the organisational context of the setting and the CRE epidemiology in that setting and its catchment population. Basically, the strategy must define the target patients and the method(s) for active screening and should be integrated with IPC and antimicrobial stewardship programs.

The choice of method for active CRE screening should be agreed with the local IPC Team, taking into account different variables (prevalence of colonization, types of circulating carbapenemases, wards with higher incidence and severity of infections caused by CPE, ease of isolation of positive and presumptive positive patients, access to Clinical Microbiology laboratory, overall costs). Moreover, it must be emphasized that active surveillance is one of several interventions within the multifaceted programs for the prevention of transmission of CPE, and the impact of active screening will depend not only on its quality but also on its contextualization within a complex bundle.

The most recent report of ECDC [45] about the effectiveness of infection control measures to prevent transmission of CPE stated that no study assessed screening/surveillance as a single intervention, but the authors concluded that there is evidence to suggest that active rectal screening/surveillance on admission to hospital or a specific ward and during an outbreak can effectively limit and prevent the spread of CPE. The ECDC document recognizes among other most effective interventions patient isolation, patient cohorting and application of contact precautions. All these measures are usually applied when a patient is diagnosed as colonized and/or infected by CPE. Molecular methods for CPE surveillance can reduce TAT for the detection of colonized patients by 48–72 h, compared to traditional culture-based methods. Although there is still limited data available regarding the impact of rapid surveillance methods in prevention of transmission of CPE, it is likely that identification and isolation of carriers 2–3 days earlier could lead to reduction in the transmission risk and a more efficient use of infection prevention resources. Moreover, rapid screening methods could make it easier to adopt the strategy of pre-emptive isolation of patients on admission until the report of a negative result, another measure considered effective by the ECDC paper.

As suggested by Lau et al. [56] in their recent review about CPE screening methods, the universal use of NAAT-based assays for surveillance swabs may not be affordable because of the increased costs, but the use of molecular rapid methods may be advisable if applied to the screening of high-risk patients (e. g. returning from areas of endemicity, transferred from LTCFs, or having had an extensive exposure to carbapenems). The authors also stated that the ideal molecular assay for most epidemiological contexts should include at least KPC, OXA-48, NDM and VIM-encoding determinants. Moreover, samples with negative or indeterminate results should be tested with a sensitive culture-based method and suspected colonies should be subjected to a confirmatory test for carbapenemase activity.

Screening for detection of CPE colonized patients is a fundamental part of CPE infection control programs. The optimal modality for surveillance strategy and methods should be evaluated by each institution considering several specific features. Culture-based screening methods can easily be implemented by most clinical microbiology laboratories and still represent the fundamental backbone of CPE active surveillance, providing the possibility to detect all types of carbapenem-resistant organisms, to perform phenotypic susceptibility testing, to collect and store the strains. On the other hand, long TAT (at least 24 for positive results) could be considered suboptimal for high-risk patients. Use of new generation of rapid-easy to use NAAT-based assays with high levels of sensitivity, specificity and very quick TAT, although still characterized by a high direct acquisition cost, may lead to more timely detection of colonized patients, thus improving the overall performance of the CPE prevention and also reducing costs related to hospitalization of patients with colonization and/or infection caused by CPE.

## Common questions about active screening for CRE, and consensus-based recommendations

### Which settings require active screening for CRE, and which patients should be screened?

It was agreed that active screening is a cornerstone in the control of CRE, including in high-burden settings. In these settings, the focus needs to be on patients at highest risk of CRE infections and of causing transmission to other high-risk groups. From an epidemiological point of view, considering the current risk factor stratification, the populations that should be targeted for CRE screening on admission to acute-care hospitals are: i) patients admitted from long-term care and rehabilitation facilities; ii) patients who are transferred directly from another acute-care hospital; and iii) patients admitted from the community with a history of hospital admission within the last 12 months [43]. From a clinical point of view, all patients in whom colonization could represent a risk factor for invasive disease should be screened on admission and then weekly during hospital stay. Severely immunosuppressed subjects, critically ill patients and patients exposed to major surgery should be the first to be included in a screening program. Accordingly, with this indication, ICUs, Transplant Units, Hematological Units, major surgical and Infectious Disease units represent the preferential setting for targeting active screening. Considering the heavy involvement of long-term care facilities in CRE dissemination in endemic settings [20, 57, 58], active screening as part of an IPC program is also recommended for all patients admitted to these structures.

### What is the role of REU-CMA?

Currently available CPE infection control guidelines do not suggest which is the most appropriate method to apply for active surveillance because of the lack of strong evidence defining the optimal method.

Although having high acquisition costs compared to conventional culture, REU-CMA seem an appealing method for CPE screening, due to the fast TAT, high sensitivity, and opportunity for information on the resistance mechanism which can be relevant for the purposes of IPC strategies and antimicrobial stewardship.

From the IPC point of view, the availability of REU-CMA could provide rapid information on colonization status, allowing to retain contact precautions only for colonized patients and to rapidly release preemptive contact isolation for patients who are not colonized. This can help to reduce the risk of inter-patient spread in high endemicity settings, due to failures to comply with long-lasting (2–3 days) preemptive contact isolation and can also be cost-effective by saving the resources required for long-lasting contact isolation with all new admissions. The additional cost of materials, disinfection and extra nursing time per patient due to preemptive contact precautions for a range of antibiotic resistant organisms, has been estimated to range between 1.27 to 4.1€ per hour [59–61]. In settings with a 10% prevalence in the high risk population, this could mean that 90% of the patients will be unnecessarily put in preemptive contact precautions waiting for the result of the screening test at a cost between 61€ to 197€ per patient if the test is performed using a culture-based technique. Early identification of patients suspected of being colonized with and CPE combined with rapid implementation of contact precautions is the cornerstone of an efficient IPC strategy [45]. Moreover, the rapid feed-back provided by REU-CMA can be particularly useful in the management of outbreaks.

From a clinical point of view, REU-CMA could be very useful to target any empirical/ pre-emptive therapeutic approach in severely ill patients at risk of CRE infection. Indeed, a rapid recognition of the carrier status and the characterization of the genetic pattern of resistance enables appropriate treatment to be targeted as early as possible.

Finally, it should be noted that REU-CMA can also be amenable to near-patient testing. This option could be particular interest in health care settings where an on-site clinical microbiology laboratory is not available, and screening samples must be sent to an external facility for processing, a situation that is often encountered in long-term care/rehabilitation facilities. Indeed, in these cases, implementation of a REU-CMA on-site, which can be handled remotely by a clinical microbiologist, can save considerable time in the identification of CRE colonized patients that require contact isolation and dedicated rehabilitation pathways.

The choice of inclusion criteria of the samples on which the molecular test could eventually be used should be shared with the local IPC Team, considering different specific local variables (prevalence of colonization, high risk wards, possibility to apply presumptive isolation) in order to optimize the clinical and economical impact.

### How should we manage information and patients in relation to carriage status?

At the moment of diagnosis of carrier status, it is mandatory to give all information to the patient, the family and the caregivers, in order to ensure that the best practices and the proper use of personal protective equipment during hospitalization are adhered to. On discharge, all information about patient status including the condition of CRE carrier must be reported to the general practitioner or to the long-term care / rehabilitation structure that will receive the patient. It is necessary that all hygienic standards are respected, and the adherence to hand washing practices is maximized, both at home and in long-term care facilities.

In order to prevent and reduce the spread of CRE, general, organizational and clinical care measures need to be put in place. For every patient admission, each hospital must adopt a flow chart for infection prevention and control (IP&C) of CPE. It has to set up the early recognition of individuals who may be colonized or infected, organize the early isolation of suspected and laboratory-confirmed cases and ensure early diagnosis of suspected cases and contacts during hospitalization by adopting a systematic screening programme.

## Conclusions

Continuing transmission of CPE in Italy and a number of other European countries remains a cause for grave concern. This position paper provides information regarding the background to the problem and a brief overview of current guidance documents, with particular respect to the role of active surveillance. While it is clear that active surveillance for carriage of CPE remains an important aspect of the overall “bundle approach” to containment, evidence is scanty regarding the precise role of screening, types of patients and clinical units to be screened, and method of screening including the role for REU-CMA. While we have attempted to provide some pragmatic advice on active surveillance in a high burden country, we also wish to highlight the importance of further research into this topic.

## Data Availability

Not applicable.

## Abbreviations

CDC: Centre for Disease Control and Prevention
CPE: Carbapenemase-producing Enterobacteriaceae
CRE: Carbapenem resistant Enterobacteriaceae
ECDC: European Centre for Disease Prevention and Control
ESCMID: European Society of Clinical Microbiology and Infectious Diseases
ICU: Intensive care units
IPC: Infection prevention and control
KPC-Kp: *Klebsiella pneumoniae* strains producing KPC-type carbapenemases
LTACH: Long-term acute care hospitals
LTACRF: Long-term acute-care rehabilitation facility
MDR: Multidrug resistant
MDRO: Multi-drug resistant organisms
MIC: Minimum inhibitory concentration
NAAT: Nucleic acid amplification technology-based assays
REU-CMA: Rapid/easy to use commercial molecular assays
TAT: Turn-around time
USD: United States Dollars
